# Agency of depressed adolescents: embodiment and social representations

**DOI:** 10.1080/17482631.2018.1564516

**Published:** 2019-01-20

**Authors:** Jan De Mol, Ann D’Alcantara, Barbara Cresti

**Affiliations:** a Psychological Sciences Research Institute, University of Louvain, Belgium; b University Hospital St-Luc, Faculty of Medicine, University of Louvain, Louvain-la-Neuve, Belgium; c Economics School of Louvain, University of Louvain, Belgium

**Keywords:** Human agency, depression, embodiment, social representations, psychotherapy

## Abstract

**Purpose:** Major depression is becoming more common among adolescents. Most research into major depression disorder focuses on intrapersonal and interpersonal processes, but the importance of sociocultural factors is less investigated. This study explores the role of social representations in the construction of adolescents diagnosed with major depression. The researched was informed by the concept of human agency and Social Relational Theory.

**Method:** Interviews were conducted with fifteen hospitalized adolescents diagnosed with a major depression disorder using a semi-structured interview schedule. The research question was: What are the social representations about being a normal person that influence depressed adolescents and their lived experiences of having major depression? Transcripts were subjected to Interpretative Phenomenological Analysis.

**Results:** Five superordinate themes emerged out of the data: (a) Depression means personal failure; (b) Feeling bad is not allowed and is not normal: in fact, depression doesn’t really exist; (c) You are obliged to have an intimate relationship, otherwise you are not normal; (d) It is important to have future projects for personal and social well-being; (e) Being socially well integrated is normality.

**Conclusions:** Clinical and therapeutic implications are discussed.

## Introduction

The prevalence of major depression in adolescents has increased in recent years. In the USA the prevalence increased from 8.7% in 2005 to 11.3% in 2014 (Mojtabai, Olfson, & Han, 2016) whereas in Europe, depending on the specific country, the prevalence ranged from 7.1% to 19.4% in 2012 (Balázs et al., 2013). Major depression disorder in adolescents is characterized by constantly having a depressed and irritable mood, a diminished interest in activities, feelings of worthlessness and guilt, thought and concentration problems, and recurrent thoughts of death and suicide (Weis, 2013). Most research into adolescent major depression focuses on intrapersonal processes, such as how processes of rumination facilitate the development of depression, or interpersonal processes, such as how social information processing, like peer rejection and victimization, enhances depression (Nolen-Hoeksema & Hilt, 2009). However, the importance of the social discourse and sociocultural factors regarding adolescent major depression is a gap in this literature (Cicchetti & Toth, 2009). Moreover, most research on adolescent major depression has taken the perspective of adolescents as victims of the disorder rather than human agents who construct their own lived experiences of the condition and context of major depression.

The purpose of this study was to investigate the role of social representations in adolescents’ construction of major depression in a sample of adolescents hospitalized with a major depressive disorder. The perspective of *human agency* (Kuczynski, 2003) is the central concept that informed this research. In order to define the research objectives, the following literature is reviewed: existing qualitative research into the subjectivity of adolescents with depression, Social Relational Theory (SRT) including the concept of human agency (Kuczynski & De Mol, 2015; Kuczynski & Parkin, 2006), and social representations theory (Jovchelovitch, 2001).

The research question that guided this research was: What are the social representations about being a normal person that influence depressed adolescents and their lived experiences of having a major depression? The research objective was informed by a dominant discourse in Western society that having a psychiatric disorder, including major depressive disorder, means having an illness or pathology and not being normal (Szasz, 2011). The word *disorder* exemplifies this discourse. Consequently, social representations of normality influence lived experiences of having major depression. Current psychotherapy and clinical interventions focus on the agency of clients diagnosed with a psychiatric disorder. Hence, psychotherapeutic and clinical implications of this research will be discussed.

### Current state of the art

Most qualitative research into adolescence major depression focuses on adolescents’ perspectives about what it is like to live with a major depression and the effects on family and social life. Adolescents with depression live constantly in a world filled with fear, associated with a fear to return to bad feelings even when the depression is under control, a fear of not being able to survive bad feelings, and a fear of not getting professional help in an efficient way (Woodgate, 2006). These constant bad feelings also influence adolescents’ identity development (Kuwabara, Van Voorhees, Gollan, & Alexander, 2007). Major depression seems to interrupt the development of a constructive sense of identity because these constant bad feelings do not correspond with how adolescents with depression perceive their own person. Consequently, adolescents with depression seem to alienate from their own person. These processes of self-alienation can also lead to social alienation (McCann, Lubman, & Clark, 2012). Adolescent major depression has effects on family and peers. Adolescents may perceive that their parents, siblings, and peers have difficulties understanding what it means to live with major depression and consequently are not always able to support them in a constructive way. Social support is a very important resource for adolescents to cope with a depressed mood. In sum, qualitative research into adolescence major depression focuses only on subjective experiences and influences on family and social life. But subjective experiences are also influenced by the social context of meanings in which the depressed adolescent is embedded. Moreover, research also indicates the influences of social factors on adolescent development (Lerner & Galambos, 1998).

A major theme in adolescent development literature regards the process of separation-individuation (Mahler, Pine, & Bergman, 1975), including identity development in the social world outside the family. Adolescents are in a process of taking space from their own family in order to construct a social identity outside their family. Consequently, adolescents are confronted with social complexities in their social world and these complexities also influence their identity development. To date, there is no research that has investigated sociocultural influences on adolescence major depression. Our research focuses on the importance of the social discourse and in particular by addressing our participants, hospitalized adolescents diagnosed with a major depression, as active agents.

### Perspective on agency of depressed adolescents

Social Relational Theory (SRT) (Kuczynski & De Mol, 2015; Kuczynski & Parkin, 2006) stresses the universal aspect of agency, which refers to the ontological assumption that all human beings, including adolescents with depression, are agents. Moreover, within SRT all humans are equally agents. Agency means that each human being is considered as an actor, with the ability to make sense of the environment, initiate change, make choices, and resist demands (Kuczynski, 2003). For analytical purposes Kuczynski (2003) partitions the concept of agency into autonomy, construction, and action. These components of agency jointly consider the complexity of interdependent motivations, cognitions, and actions that are coordinated in a single process. In the current research project, the focus was on the concept of construction. Construction refers to a person’s capacity to make sense of their own behaviours, the behaviours of others and to construct new meanings from these experiences. The process of sense making involves both cognitions and emotions. In order to make the process of meaning construction more explicit, the concept of embodiment is of central importance. Each human is an active agent with a particular kind of lived body (Andersen, 2007; Overton, Mueller, & Newman, 2008). Embodiment refers not only to physical structures but also to the engagement of the body in the sociocultural world (Overton, 2006).

“The *body as form*, represents the holistic integration of the biological dimension of life, the *body as lived experience actively engaged* represents the integration of the psychological person, and the *body actively engaged in and with the world* points to the integration of the sociocultural and physical context. Thus, embodiment entails the synthesis of how we, as active agents (psychological persons), influence and are influenced by our biological and sociocultural worlds.” (Kuczynski & De Mol, 2015, p. 330)

The concept of embodiment signifies that we, as equally human agents, are being influenced through our body, and it is our body that forms the bridge between the personal, relational, and sociocultural level (Varela, Thompson, & Rosch, 2016). However, individual differences between humans exist that affect the quality of expression and effectiveness of agency. The process of meaning construction takes diverse forms related to specific individual resources (the particular biological body) and social resources (sociocultural context). Adolescents with depression have a particular biological body and are influenced by particular sociocultural discourses. Consequently, these two factors influence how adolescents with depression construct meanings of their lived experiences as active agents.

Current theorizing about embodiment focuses on the interplay between embodied experience and influences of social representations (O’Connor, 2017). In our research the concept of social representation was chosen to investigate influences of sociocultural factors on processes of meaning construction of depressed adolescents. A social representation is the ensemble of thoughts and ideas that exists in a social community regarding a specific phenomenon (Jovchelovitch, 2001). In other words, social representations are common thoughts or common sense knowledge co-constructed in dialogues and expressed in verbal and overt behaviour of actors within a sociocultural context. Objectification regards an important step in the construction of social representations (Wagner et al., 1999). In social communication people develop their own interpretations of phenomena by constructing a socially shared knowledge in a specific form, that is, a metaphor or social representation that captures the complexity of the phenomenon. This symbolic knowledge regards the creation of shared understandings as the semiotic space in which we live and is of central importance to human social life. Social representations are constructed in dialogues but it is through objectification that they become realities that exist outside of our social communication. Consistent with social representations theory, social representations were investigated in this research by taking a realist epistemological position. Social representations exist independently of the participants’ and researchers’ views and knowledge about it. A realist epistemological position assumes that by doing inductive research knowledge can be generated about something that exists in the real world (Willig, 2013).

In conclusion, the rationale of this study and the research question being addressed may be summarized as follows: what are important social representations about being a normal person that influence depressed adolescents’ lived experiences of being depressed. Consistent with the concept of agency, embodiment, and social representations theory, the importance of the social discourse influencing adolescents’ lived experiences is studied. This perspective is fully in line with current approaches in psychotherapy. Consequently, this research may inspire psychotherapists’ clinical interventions in their daily practice.

## Method

### Participants

Interpretative Phenomenological Analysis (IPA) (Smith, Flowers, & Larkin, 2009) was chosen as the method for data collection and analysis. IPA has already been used to explore social representations influencing persons’ lived experiences (Jaspal & Yampolsky, 2011; Mercer & Feeney, 2009). Purposive sampling was employed to recruit adolescents within the age range of 15 to 18 years old. The sample consisted of 15 adolescents, 9 girls and 6 boys, hospitalized for major depression at the Department of Psychiatry for Adolescents of the University Hospital of Louvain in Belgium. Consistent with the requirements of IPA, this sample size is small enough to facilitate an idiographic analysis (Smith et al., 2009). All adolescents were diagnosed with major depression by a psychiatrist and first received outpatient psychotherapy. The reasons for hospitalization included treatment-resistant depression or symptoms putting the adolescents at risk, such as suicidal ideation, school refusal including anxieties, mania, and psychotic episodes. Adolescents who participated in this research had been hospitalized for at least 4 months.

### Research ethics

The study was approved by the second author, who is chief psychiatrist of the Department of Psychiatry for Adolescents of the University Hospital of Louvain in Belgium. Because this research included therapeutic applications in line with the therapeutic program of the department, the head of the department was responsible for the research ethics, and by giving approval this research was also approved by the ethics committee of the University Hospital, Faculty of Medicine of the University of Louvain. The chief psychiatrist decided which adolescents could be interviewed, asked the adolescents if they wanted to participate in this study and explained the objectives of the study respectfully in an open and transparent way. The adolescents were completely free to decide if they wanted to participate or not. When the adolescent agreed to participate, the parents of the adolescent were contacted to get their permission. Both adolescents and parents signed the informed consent form. The audio-files of the interviews were transcribed verbatim. To protect confidentiality, all names were modified.

### Data collection

The adolescents were interviewed in the Department of Psychiatry for Adolescents of the University Hospital of Louvain. The third author, a clinical psychologist, conducted the interviews. The interviews lasted 60 minutes on average. Consistent with IPA the interviews were semi-structured, using an interview guide exploring the following topics: explanations that exist in our society regarding being depressed and being normal, adolescents’ own ideas regarding these explanations, and how these explanations might influence their own emotions and well-being. IPA focuses on participants’ lived experiences and how they make sense of these experiences. According to SRT and social representations theory, meanings are co-constructed in relationships and these co-constructions are influenced by social representations, which corresponds to the theoretical assumptions of IPA. The in depth explorations of participants’ lived experiences and semiotic dimensions of social representations are also consistent with the concept of embodiment (Jaspal & Yampolsky, 2011; Mercer & Feeney, 2009).

### Analysis

Data-analysis was performed using the different phases of IPA (Smith et al., 2009). Each interview was analyzed as a separate case. The first phase consisted of rereading the transcripts of the interviews. In the second phase, descriptive, linguistic, and conceptual notes were made. In the third phase, themes were identified by looking for patterns and connections across these notes. The fourth phase consisted of grouping these themes into subthemes and higher order themes based on similarities and differences. This process was repeated for each interview. Next, superordinate themes were constructed which were common to most participants. Last, narratives were written to explain each superordinate theme in depth.

To enhance the trustworthiness of the data-analysis, investigator triangulation was used. Each researcher engaged in the process of bracketing by discussing own assumptions with the two other researchers. Data analysis was completed by the third author. The first author, clinical psychologist-psychotherapist and researcher, evaluated the different steps of the analysis and the third author repeatedly reviewed the analysis. When the superordinate themes were constructed, the narratives of each superordinate theme were discussed with the second author, and based on these discussions superordinate themes could be renamed. Finally, the superordinate themes were discussed with the adolescents to evaluate their pertinence and relevance.

IPA is a version of phenomenological methodology that takes a critical realist epistemological position by accepting the impossibility of gaining direct access to participants’ life worlds (Willig, 2013). The researcher is engaged is a double hermeneutic approach (Smith et al., 2009) by trying to make sense of the participants trying to make sense of their lived experiences. Larkin, Watts, and Clifton (2006) state that IPA “positions the initial ‘description’ in relation to a wider social, cultural, and perhaps even theoretical, context. This second-order account aims to provide a critical and conceptual commentary upon the participants’ personal ‘sense-making’ activities.” (Larkin et al., 2006, p. 104). Consequently, superordinate themes produced by IPA are researchers’ interpretations of participants’ experiences. When the superordinate themes were discussed with the adolescents, they stated that the themes expound in depth their lived experiences.

## Findings

Five superordinate themes emerged out of the data. Each theme describes current social representations in adolescents’ social community regarding what it means to be a normal person and how these representations influence adolescents’ lived experiences of being depressed. In particular, participants focused on social expectancies about normality and their normative character. Adolescents with depression seem to experience influences of social representations as a constant pressure that intensified their lived experience of being depressed. Social representations may have a normative or binding character but are not normative or binding by definition (Jovchelovitch, 2001). Nevertheless, our participants as active and interpretive agents experienced these social representations as oppressive due to their normative character. In line with the inductive approach of IPA, the objective of each verbatim is to underpin the narratives of the themes and not to confirm them (Smith et al., 2009).

### Theme 1: depression means personal failure

The most common theme, reported by all the participants, was that depression was equated to personal failure. Participants explained that they as adolescents were becoming more and more sensitive to other persons’ views and opinions about them including the opinions of peers, teachers, parents, and other adults. The pressure they felt has to do with ideas that exist in the society about failing. A dominant idea was that when you cope with an issue in an efficient and proper way, the outcome will be good. This means also that you always have to know, and that you can know, how to cope with an issue. And if you are depressed, this means you have personally failed to cope.

Girl (16 years): When they told me that I had a depression, I was really shocked… In current society there is a real problem. Everybody wants to show that they are superior. You have to be able to cope with everything, you always have to find the solution. I don’t understand it, they [the society] are hurting people because you have people who are more vulnerable than others. It makes me so sad.

The social discourse that people can always know how to deal with something, also means that failing is not accepted. Not being able to cope with something is not allowed. Failing can only be accepted when the person is successful in other circumstances. Consequently, when a person fails, this means that the person has failed, or that the failure is a personal responsibility.

Girl (15 years): I worked a lot at school, it became something obsessive. But I had bad results. I didn’t understand it, because I worked a lot, and when you work a lot it’s normal that you have good results. I had the impression to be nobody, I felt inferior to others. You put that pressure on your own person but without any result. In fact, it was especially me who put that pressure on myself.

Many participants talked about expectancies that exist in society regarding school performance, and in particular how this influences their sense of agency. Failing at school became a source of shame, disappointment, and demotivation. Consequently, participants stated that they were losing their sense of being active agents and their ability to do something with their own lives.

Girl (18 years): I failed at school. This has a real impact on your person. I saw a lot of adolescents at school who fail, and all of them had bad feelings. All those teachers are always saying that you have to get good marks, because this is important for your future. So, they were saying that I wouldn’t have a future. This really broke me.

Some adolescents also stated that their anxiety about failure was linked with an anxiety about disappointing other people and in particular their own parents. Adolescents with depression seemed to be very occupied with other persons rather than with their own person. This engagement towards others enhanced their anxiety about failure.

Boy (15 years): I always intended to do what I can so that my parents could be proud of me. But when I had bad grades, I was really afraid to disappoint them. This was always in my head, that I would disappoint them when I had bad grades. It was always there, in my head, but I didn’t know what to do with it. Totally not funny.

As active agents, adolescents with depression also tried to cope with this social pressure. For example, one adolescent explained how his ability at playing Internet games was important to him. Due to the difficulties of obtaining success in real life he was looking for succeeding in the virtual world. He explained that internet games can be helpful to receive social recognition because in these games you can meet the social expectations.

Boy (18 years): I did a lot of bullshit, I was well known, not for my bullshit, but because of my competence as Internet player, that everybody admired. I was hyper well known in Blablaland, no clan stood against me, plenty of people came to me, others begged them and I accepted them. All that stuff, I even received gifts. Yes, that gave another feeling.

This adolescent explained a way to do something with the constant social pressure, but also that this way of coping did not resolve the problem for him. Feelings of unhappiness remained present but by visiting the virtual world his processes of rumination could decrease for some time.

In summary, it was apparent that adolescents were alert to social representations that depression signifies personal failure and this meaning adversely affected their sense of well-being.

### Theme 2: feeling bad is not allowed and is not normal: in fact, depression doesn’t really exist

All participants expressed the need and even the necessity to hide their sadness from others, due to their anxiety of being judged and socially excluded. Talking about feelings of unhappiness was difficult in their society because most people do not respond or run away from it. People seem to have difficulties responding to or dealing with feelings of sadness of others. Participants reported that they could not express their negative emotions because they feared various negative consequences including social exclusion, isolation, and superfluous advice that they do something about it or cope with it.

Girl (18 years): I cannot talk about my sadness, in fact, I don’t dare to talk about it, because then you are considered as a weak person. I see that some people feel pity for me, but they don’t talk to me, they prefer to run away because they are afraid and do not know how to react to someone who is sad.

Girl (17 years): I started to feel very bad, and more and more I isolated myself, and my friends never asked me what was happening, never, they only told me that I had to stop with it, and at the end of the school year they told me: “Listen, you are no longer a friend, we do not want to hear from you, you have to get out of our lives”, and so it is. And this really hurts me.

Perceptions that one should not express negative emotions in society gave rise to a process of social isolation for participants. This process of social isolation was characterized by ambivalent feelings. Participants explained that on the one hand they feel the necessity to share their emotions with others, but on the other hand they felt it was impossible to do this. Consequently, they felt caught up in a vicious circle which made them feel alienated from themselves and from of their social world.

Boy (15 years): I’ve had enough, they don’t listen. And when you become lonely, then you distance yourself from others. I don’t have friends anymore… Because many times I’m alone, I have nobody to talk to and I can deal with it… No, I turn other people down.

This process of social isolation is even more facilitated by another social representation that diminished the significance of their depression. All participants described and denounced how others in their social contexts trivialized their experiences of depression. By trivializing and minimizing adolescents’ sorrow, the adolescents’ experience was not taken seriously. Some participants went further and perceived that others in fact questioned the existence of their depression. Participants described two kinds of social discourse that undermined the reality of depression. The first regards discourses that the prevalence of depression is exaggerated in society. Everybody has bad moods or feels down sometimes, but this does not mean that one has the disorder of major depression. Consequently, the depressed person perceives that their horrible experience is negated and their use of the word “depression” is an overstatement of what is really going on.

Girl (15 years): Most people say it’s just a lack of energy, it’s not a real illness. Some other people say that it can happen, but then they say also: “Yes, it can happen, but make it easy, there is much worse than that”. But when they are saying that, it feels as if they don’t recognize my suffering.

People may be attempting to minimizing the depression to help the adolescent but in actuality their comments may have the opposite effect. The depressed adolescent may feel that their authentic experience was being ignored and consequently may feel increased sadness and social alienation from significant people in their lives.

The second explanation is linked with the traditional linear-causal way of thinking in Western society. When you feel bad, you need to have an external explanation for why you have these feelings, because the fact that you feel bad must be caused by something. Participants stated that they often received the question: “*Why* are you feeling so bad?”. Adolescents shared that they cannot give a constructive answer because they do not know why they have these feelings. They could not give explanations because there were no specific causes for them. Due to the inability to provide a real explanation regarding the causes, their feelings and depression are not recognized by others.

Girl (18 years): If someone asks you what is wrong with you, and you don’t really know what is wrong with you, you say, “I don’t know”. When I say, “I don’t know”, that person thinks that there is nothing wrong, because when there is really something wrong, then you will know what is wrong.

Some of the participants admitted at the start of the interview that they had a depression, but during the interview they denied it, although they talked in a profound way about the complexity of being depressed. Their denial of their own feelings appeared to be a way to cope with the non-recognition of depression in society. By denying their depression, they presented themselves as normal people, but could simultaneously explain the complexities with which they are confronted.

Boy (15 years): I don’t have a depression. People laugh at depressed people. Or they laugh at them, or they feel sorry for them. I don’t like that. Regarding myself, I don’t have a depression, so I don’t care.

### Theme 3: you are obliged to have an intimate relationship, otherwise you are not normal

Many participants perceived tremendous social pressures for them to have an intimate personal relationship or romantic partner. There were two main reasons that participants stressed the importance of having an intimate relationship. First, it was important to be in a romantic love relationship in order to be able to express one’s own emotions in a secure context. Although parents and other family members stay important, participants perceived that there was an expectation for adolescents to undergo a process of separation-individuation, which means that they must create more distance from their family to construct an identity in their own social world. Consequently, having other intimate relationships that can serve as sources of intimacy and social support are a part of normal development. Constructive social sharing of emotions was facilitated when adolescents with depression have feelings of security and closeness in the relationship. Moreover, sharing emotions in a secure context had a constructive influence on their own wellbeing because this meant that they were allowed to have these emotions.

Boy (16 years):It’s very good that I have a girlfriend. Then you have someone who is very close to you and whom you can tell things, someone you can really trust. Having trust, and to trust someone, that’s not something to be taken lightly. This is so important for me.

The second reason why intimate relationships were important for our participants was because having intimate romantic relationships was perceived to be a requirement of their friendship networks. Although some participants talked about sharing emotions in friendships, they thought it is much more constructive for them to do this in romantic relationships. Participants explained that when they are not in an intimate relationship, they are perceived by others as not normal. A social representation in their social community seemed to be that you have to be in an intimate romantic relationship otherwise you are not normal. Moreover, a person who is not in an intimate relationship has to explain why, and these reasons have to be strong and well-argued. For adolescents with depression it was very difficult to give well-argued explanations, because they were perceived by others as not normal and their lack of a romantic relationship partner was a direct consequence of their depressive disorder.

Girl (18 years): To be loved by someone is very important. It’s a personal satisfaction because you receive love from another person and you feel well because you know that person is supporting you. This is what everyone thinks. Me too… I don’t have a boyfriend now, I think it’s due to my depression. I think that people think that I’m not capable to have a relationship. I can understand that nobody wants to have a relationship with me now, but I miss it.

Other adolescents explained the massive impact of losing a partner on their own significance as a person. Constructing and maintaining an intimate relationship was an important achievement that others valued and consequently was impactful for adolescents in developing a social identity. When this relationship fails, adolescents lose their self-esteem and social identity. Adolescents with depression were very sensitive to comments of others and perceived that the termination of a romantic relationship termination was yet another failure that contributed to feelings of sadness and low self-worth.

Girl (17 years): Yes, I was really in love. And then he leaves you. When love hurts, you get more depressed because you are losing self-confidence.

### Theme 4: it is important to have future projects for personal and social well being

The participants of this study also discussed concerns about their future plans and achieving a state of well-being. They had a persistent sense of anxiety that their bad feelings will not stop, and could hinder their ability to build a future. Their concerns about their future were exacerbated by social expectations of others. A social representation in their society seems to be that a person needs to have future plans otherwise this person is not able to develop a real personal life. This representation enhances the cycle of adolescents’ negative emotions because of the intensification of feelings of failure.

Girl (16 years): Everyone, even the therapists here, always asks me what my future plans are, like, what fields of study that I wish to follow, what profession that I would like to pursue. But I don’t know. And that I don’t know it, makes me even more stressed. I’m really afraid about my future, I don’t know what will happen.

The social expectation to have future plans also seems to be linked to the importance of having good school results and academic achievement in our society. Schooling and education were associated with the personal development and future of each individual. The evidence in the social discourse seems to be that having good personal development at school will have an important and even a determining influence on adolescents’ future life. Consequently, when academic achievement is problematic, adolescents said their future was in danger.

Boy (15 years): You have so much homework to do. You are overloaded with work, and then you get bad feelings. They always say that it is important for my future. If you don’t succeed then you cannot realize what you want. I don’t have faith in the future anymore.

In describing social expectations for his achievement, this participant emphasized the messages conveyed to him by his parents. Parents are also influenced by social representations about the importance for their adolescents of having future projects. Some participants explained that their parents tried to help them to get them back to being happy by stimulating them to act in an efficient way. Consequently, parents tried to help their adolescent but appeared unaware of their influence on the construction of negative emotions in their adolescent.

### Theme 5: being socially well integrated is normality

Many participants identified a social representation regarding the perceived requirement to have the knowledge and the skills regarding how to integrate, in a normal and efficient way, in the social environment. Consequently, depressed adolescents perceived it to be self-evident that they should know how to behave like a normal person in their social contexts.

Boy (17 years): Recently I had a discussion with my brother. I took always the tram to go back home after school. When I was on the tram, I was always very nervous, because others were always talking and laughing together, and I didn’t know how to behave myself. And when I told it to my brother, he said: “But don’t worry about it, just do and say what you want. I cannot explain what you have to do, but just act normal, don’t worry.” I know that he wants to help me, but people don’t seem to understand that it is very difficult for me to know how I have to behave myself when others are present.

All participants completed social skills training in their department of the psychiatric hospital. They explained that it was very interesting and enriching for them, but also difficult due to the complexity of integrating these skills in your own life. The most important lesson for the adolescents concerned the acceptance of not always knowing what it meant to act in a socially competent way. An important objective of social skills training is not to create the illusion of always knowing what to do and how to act within social contexts.

Boy (15 years): I very much like that training. I also learn that you cannot always know which exact skill you need to use. It’s difficult, but I feel better now.

Participants felt that this particular lesson of social skills training was valuable because it attacked the social representation that becoming well socially integrated is a normal, easy, and evident process. However, constructing a social identity that included social skills and social competence seemed to be important to cope with depression. Two participants, who were finishing their therapy and hospitalization, explained the importance of being well socially integrated to handle their depression in a constructive way. By becoming part of a community or a group, feelings of depression are diminishing because a social identity can be reconstructed.

Girl (18 years): I get along with people, I notice that people love me, I’m really ready to improve social interactions, and this, this helps me a lot, I’m much happier now.

## Discussion

The analyses revealed how adolescents who are depressed act as interpretive agents of their own experiences and social environments. Adolescents were able to describe social discourses concerning their depression and how these discourses impacted on their person and lived experiences of being depressed. The different social representations discussed in the themes are experienced by our participants as obligations imposed in their social community to which they had to satisfy. When these obligations are not fulfilled, the lived experience of not being a normal person is intensified which feeds into processes of depression, such as sad mood, social isolation, and rumination processes. Consistent with the concept of embodiment, not only the biological body but also social representations affect the embodied lived experience of being depressed. Our participants were hospitalized adolescents diagnosed with a major depression. The reality of being hospitalized might have influenced participants’ lived experiences of the obligatory and normative character of social representations. Nevertheless, our hypothesis remains that these social representations have an oppressive effect on depressed adolescents lived experiences of being depressed, and that hospitalized adolescents, as active agents, can better sense and explain this pressure due to their hospitalization.

### Clinical and psychotherapeutic implications

Qualitative research is highly important for clinical and psychotherapeutic practice because by exploring in depth participants’ lived experiences, fundamental knowledge about a clinical phenomenon like depression is gained. Consequently, knowledge co-constructed in dialogue with participants about a clinical phenomenon is instructive for clinical and psychotherapeutic practice. Moreover, qualitative researchers approach participants as active agents. This approach is in line with current evolutions in family therapy, more in particular collaborative-dialogical psychotherapeutic practices (Anderson, 2012) and narrative therapy (White, 2011).

Although doing qualitative research is not the same as doing psychotherapy, many participants stated after the interview that it was an interesting and enriching experience for them that helped them to better understand their own feelings and thoughts, regarding what their body is telling them. Research into emotion regulation (Rimé, 2009) demonstrates the connection between the body and social processes, and that by the social sharing of emotions a persons’ insides is reconnected to the social discourse and this process regulates emotions. Based on this research, emotion regulation may be improved when adolescents with depression share their emotions during therapeutic practice, and the therapist discusses in dialogue with the adolescent current social representations in order to contextualize adolescents’ emotions within the social discourse. This approach is in line with narrative therapy (White, 2011) which stresses the importance of human agency in the therapeutic process. In narrative therapy clients are not regarded to consist of the problems that they present to the therapist. People enter therapy by telling only their problem-saturated stories. The objective of narrative therapy is to co-construct with clients as active agents other narratives next to the problem-saturated stories. Based on our research, by externalizing social representations about normal life, other narratives next to the problem-saturated stories can be co-created with adolescents with depression because what the adolescents feel and think is influenced by social representations of normality. Consequently, their embodied experience and meaning construction are valuable and not alienated as they are connected to the social discourse. By discussing social representations in therapy, the social world outside the therapy room can be brought inside the therapy, which facilitates the reconstruction of adolescents’ sense of agency in their social context.

Constructing and reconstructing a sense of agency is a bidirectional and interpersonal process (De Mol, Reijmers, Verhofstadt, & Kuczynski, 2018; Kuczynski, 2003). Within SRT, parents and children are approached as equally human agents, which means that both children and parents contribute to the construction of their relationship and the other person’s sense of agency. This approach is consistent with collaborative-dialogical therapy (Anderson, 2012), in which both client and therapist are considered as equally agents. Consequently, an important therapeutic implication of our research concerns also the knowledge that was co-constructed between the adolescent and the researcher, which may help the construction of the therapeutic alliance in psychotherapy. Within collaborative-dialogical therapy the therapist takes a not-knowing position, which means that the therapist does not take an educational stance. But this stance does not mean that the therapist knows nothing. Taking a not-knowing position means that the therapist does not know the clients’ experience or how to solve it but does know important issues that can enhance the co-construction of knowledge between the client and the therapist as equally agents in the therapeutic process. The knowledge that emerged out of our research is important for the therapist, because it concerns knowledge about the importance of the social discourse, co-constructed during a qualitative research process with depressed adolescents. This knowledge can provide the therapist with useful ideas to construct a therapeutic relationship that helps adolescents with depression act as active agents in their process of reconstructing a social identity.

### Limitations

This research has limitations. First, IPA was chosen as the method, which means that the focus was simultaneously on the content and the process of social representations influencing adolescents’ lived experiences of being depressed. Additional research using discourse analysis could enrich the content complexity of social representations that were found in this research. Second, although the results were discussed with the participants to enhance the trustworthiness of data collection and data analysis, no methodological triangulation was used. Only semi-structured interviews were conducted and it is acknowledged that focus groups would have been interesting given the research question focusing on the social discourse. Third, all researchers involved in this project have a clinical orientation. Although processes of personal reflexivity were done to explore social representations important for each researcher, it would have been interesting to involve a non-clinical researcher to add other important perspectives, e.g., developmental psychological or social psychological perspectives.

To conclude, we think that our research can be fruitful for therapists working with adolescents diagnosed with a major depression and adolescents with depression themselves, including their family and social environment. The most important issue that we want to stress is that depression is also meaningful. Persons who have experienced a depression understand that the depression will always make part of their life. Consequently, it is also useful and important as active agents to learn constantly important issues from your depression.
